# MiR-34c promotes hepatic stellate cell activation and Liver Fibrogenesis by suppressing ACSL1 expression

**DOI:** 10.7150/ijms.51589

**Published:** 2021-01-01

**Authors:** Binbin Li, Jiaxuan Liu, Xuan Xin, Lifen Zhang, Jiaming Zhou, Chunyan Xia, Weijian Zhu, Hongyu Yu

**Affiliations:** 1 Department of Pathology, Changzheng Hospital, Navy Medical University (Second Military Medical University), Shanghai 200003, China.; 2 Department of Pathology, No. 960 Hospital of People' Liberation Army, Jinan 250031, China.; 3 Department of Pathological Anatomy, Nantong University, Nantong 226001, China.

**Keywords:** miR-34c, ACSL1, liver fibrogenesis, fatty acid, lipid metabolism

## Abstract

Normally, there are multiple microRNAs involved in the pathogenesis of liver fibrosis. In our work, we aimed at identifying the role of miR-34c in the hepatic stellate cell (HSC) activation and liver fibrosis and its potential mechanism. Our results have shown that during natural activation of HSC, the level of miR-34c was increased significantly whereas acyl-CoA synthetase long-chain family member-1(ACSL1), which is a key enzyme can affect fatty acid(FA) synthesis, was decreased. A double fluorescence reporter assay further confirmed that ACSL1 is a direct target gene of miR-34c. Moreover, the inhibition of miR-34C can attenuate the synthesis of collagen in HSC-T6. In our rescue assay, ACSL1 expression was 1.49-fold higher compared to normal control cells which were transfected with the miR-34c inhibitor in a stable low expression ACSL1 cell line. While at the same time, α-SMA and Col1α expression decreased by 18.22% and 2.58%, respectively. Moreover, we performed an *in vivo* model using dimethylnitrosamine (DMN) in conjunction with the miR-34c agomir, combined with the treatment of DMN and the miR-34c agomir can increase liver fibrosis. Meanwhile, the degree of hepatic fibrosis was increased and lipid droplets reduced dramatically in rats and HSC-T6 cell treated with miR-34c mimics alone compared to untreated groups. Our results indicate that miR-34c plays an essential role in liver fibrosis by targeting ACSL1 closely associated with lipid droplets, and it might be used as a potential therapeutic target.

## Introduction

Liver disease is a major cause of death and illness around the world. Specifically, liver fibrosis is a common key pathology resulting from a variety of chronic liver diseases 1, 2. If there is no suppression or reversal of liver fibrosis, fibrosis can progress to cirrhosis which is a critical risk factor for liver cancer and a challenge for human health, resulting in 1.16 million deaths annually worldwide 3. However, no effective prevention and treatment are available due to mechanisms of liver disease have not been completely elucidated 2, 4, 5. Among liver cells, Kupffer cells, endothelial cells, and hepatic stellate cells (HSC) are involved in the development of liver fibrosis. HSC activation and proliferation represent pivotal mechanisms that trigger liver fibrosis 6-8. In recent years, microRNAs (miRNAs) have been recognized as one of the most important regulators in post-transcriptional regulation of gene expression and the differential expression of multiple miRNAs is related to HSC activation, proliferation and even apoptosis closely 9. A growing body of research indicates that many miRNA families are associated with HSC activation and the development of liver fibrogenesis by synergistically modulating certain signaling pathways 10-13. In our previous study, miRNAs were differentially expressed in animal models of liver fibrosis, as determined by chip screening. MiR-34 a/c expression is significantly up-regulated and positively correlates with the development of liver fibrosis 14. The miR-34 family has three members, including miR-34a, miR-34b and miR-34c 15. In mammals, miR-34a is located at chromosome 1p36 while miR-34b and miR-34c share primary transcription from chromosome 11q23 15, 16. There are numerous reports of alterations in miR-34 expression in cancer highlighting the importance of this microRNA family in oncogenesis. However, the role of miR-34c in HSC activation and liver fibrosis as well as its potential mechanism is still unclear.

We have initially confirmed the miR-34 family targets acyl-CoA synthetase long-chain family member-1 ACSL1 14. Members of the ACSL family include five different ACSL isoforms. ACSL1, ACSL4 and ACSL5 are present in the liver and adipocytes, whereas only ACSL1 is a key enzyme with triglyceride synthesis 17-19. In this study, we demonstrated the function and molecular mechanisms of miR-34c in hepatic fibrosis. Firstly, we isolated rat primary HSC and examined miR-34c and ACSL1 expression during natural activation of HSC. Our results showed that miR-34c was significantly up-regulated whereas ACSL1 was down-regulated. Then the double-luciferase reporter assay was used to confirm that ACSL1 was a direct target gene of miR-34c. Furthermore, inhibition of miR-34C attenuated expression of profibrotic markers and cell proliferation in HSC *in vitro*. These findings were subsequently verified in rescue assay. It has shown that miR-34c inhibition partly restores the effect of ACSL1 function.

In normal livers, HSC is quiescent and filled with cytoplasmic lipid droplets containing retinoids (especially retinyl palmitate) and triacylglycerols, accounting for more than 70% of lipids 8, 20. HSC transition from quiescent state to fibrosis, characterized by the gradual loss of lipid droplets and retinoids, is accompanied by increased α-smooth muscle actin (α-SMA) and type I collagen (Col1α) expression 21, 22. Therefore, exploring potential factors affecting HSC activation and relationship between lipid and liver fibrosis is useful for elucidating potential molecular mechanisms. Animal experiments have further confirmed that miR-34c promotes liver fibrosis and surely lipid accumulation in the liver was significantly reduced while conducting animal experiments. Moreover, *in vitro*, transfected with miR-34c mimic and inhibitor in HSC-T6, the result was consistent with *vivo* data by Sudan black staining.

In general, our findings revealed that miR-34c plays an essential role in liver fibrosis by targeting ACSL1 closely associated with lipid, and it might be used as a potential therapeutic target.

## Materials and Methods

### Cell culture and transfection

HSCs were considered quiescent on day 2 and then activated on day 14. The day before transfection, HSCs were seeded into 6-well plates and 2,000 μl of high-glucose dulbecco modified eagle medium (DMEM) containing 10% fetal bovine serum (FBS) was added. Due to the fast growth of activated HSC, the cell density was 3 × 10^5^. Cells were transfected with an inhibitor of miR-34c or with a control NC sequence using a riboMONITORTM transfection indicator. The rate of cell growth, cell status and contamination were studied 6 to 72 h after transfection.

### Immunofluorescence assay

In a 6-well plate, the slides of the grafted cells were immersed in PBS three times for 3 min each cycle. Then, slides were fixed in formaldehyde solution for 10 min, and washed with PBS three times for 1 min each, and treated with 0.5% Triton X-100 (formulated in PBS) for 20 min at room temperature. Slides were again rinsed with PBS three times for 3 min and treated with normal goat blocking serum at room temperature for 30 min. Then primary antibodies were added (α-SMA, 1:100; Desmin 1:100) and slides were placed in a wet box and incubated overnight at 4°C. The following day, slides were washed with PBS-Tween (PBS-T) three times for 3 min each. Finally, the fluorescent secondary antibody was added and the reaction was incubated in a wet box at 20-37°C for 1 h. Slides were again washed with PBS-T, three times for 3 min each, and counterstained with DAPI for 5 min. Microphotographs were taken and saved.

### RNA extraction and qRT-PCR analyses

Total RNA was extracted using the Trizol reagent (Invitrogen, USA) according to the manufacturer's protocol, and total RNA was quantified using a Nanodrop 3300 (Thermo Scientific, Wilmington, DE). 500 ng of RNA was reverse transcribed into cDNA according to the manufacturer's instructions (Takara, China) followed by amplification of cDNA using SYBR GREEN (Takara, China) with the assistance of the CFX96 Touch system (Bio-Rad, USA). ACSL1, Col1α and α-SMA and mature miR-34C relative expression levels were calculated using GADPH and U6 snRNA as housekeeping genes. The upstream and downstream primers required for qRT-PCR were designed and provided by Shanghai Biotech Co., Ltd. The primers used are showed in Table 1.

### Western blotting

The transfected cells were lysed in RIPA buffer (Biomed, China) to extract the total cellular protein. Protein concentration in the cell lysates was measured for protein using the BCA method. Depending on the size of the protein we used 5% or 10% (20-80kD) SDS-PAGE separation gels. A total of 20ul of protein was loaded and proteins were separated by gel electrophoresis and transferred onto a PVDF membrane. The membrane was blocked in PBS-T with 5% non-fat dry milk for 1 h at room temperature. The protein bands were then incubated for 4 h at room temperature in anti-ACSL1 (Thermo), anti-Col1α rabbit polyclonal antibody (ab34710), anti-αSMA (ab5694). Anti-GAPDH rabbit monoclonal antibody (ab9485) was used as an internal control. Afterwards, the membranes were incubated in PBS-T diluted rabbit secondary antibody (1:5000) (Abmart, China) for 1 h at room temperature. Protein bands were visualized using ECL luminescent solution and the images of protein bands were got using a BIORAD Flour-S Muiltilmager Gel Imager.

### Luciferase reporter assay

pMIR-REPORT-ACSL1-3'UTR wild-type plasmids (pMIR-ACSL1-WT), pMIR-REPORT-ACSL1 mutant plasmids (pMIR-ACSL1-MUT), pMIR-REPORT luciferase empty plasmids (pMIR) and plasmid containing overexpression of miR-34c (miR-34c up) or control miRNA (miR-NC up) (purchased from Shanghai Jikai Gene Chem Co., Ltd.) were co-transfected into HEK293, and the empty vector was used as control. Cells were seeded in 24-well plates before transfection, and the density of each well was about 3 × 10^5^. Fluorescent gene expression in the plasmid was observed to determine transfection efficiency after 24 h. The fluorescence of Firefly and Renilla luciferase were detected using a microplate reader.

### Construction and screening of stable HSC lines with low expression of ACSL1

According to the RNAi sequence design principle, three pairs of shRNA oligonucleotide sequences targeting the ACSL1 mRNA target sequence were designed: shR1 (GCGATAATCTGTTTCACAAGT), shR2 (GGATGACCTCAAGGTGCTTCA), and shR3 (G CCCTAGATAAAGATGGTTGG). shRCON (TTCTCCGAACGTGTCACGT) lacking homology to any known human sequence was used as a control parameter. Synthesis of the shRNA DNA oligonucleotides, annealed to form double-stranded. The recombinant plasmids were cloned into plasmid pLKD-CMV-G & PR-U6 by restriction endonucleases Age I and EcoR I and transformed into *E. coli* DH5a. The recombinant positive clones were identified by restriction enzyme digestion and sequenced. The recombinant lentiviral expression vector and lentivirus packaging plasmid were co-transfected into 293T cells (named as Lenti-ACSL1-shRNA) to obtain the lentivirus concentrate, and the virus titer was calculated. HSC-T6 cells were infected with lentiviral particles of the appropriate titer (MOI = 200) in stable cell line screening experiments. Cells that had not been effectively infected were killed by adding and maintaining 2 ug/ml of puromycin, and stably mixed stable strains were screened after 14 days. Real-time fluorescence quantitative PCR (RT-qPCR) was used to detect the change of ACSL1 gene expression in HSC-T6 cells, and the shRNA sequence with the best interference effect was screened out for further experiments.

### Animal experiments

Three-week-old Sprague-Dawley rats, weighing 50 ± 5 g, were obtained from the Institute of Zoology, Chinese Academy of Sciences. All rats had free access to standard laboratory food and water in a pathogen free animal housing facility. Twenty-eight rats were randomly divided into three groups and labeled with picric acid. The model group (#1 - #12) was only challenged with 0.1% DMN (10 mg/kg body weight, intraperitoneal (i.p.) three times per week); model group + miR-34c agomir administration group (#13 - #24), and the untreated control group (#25 - #28); The miR-34c agomir was dissolved in 100 μl of saline and then injected into the tail vein at a dose of 100 μl/ 50 g body weight every 72 h. Body weight was measured prior to adding each dose. Daily observation of rat activity and eating status was performed. Every other week, rats were randomly selected and sacrificed and the liver was extracted, weighed and fixed in formalin. Masson's trichrome and Sudan black staining was used to determine collagen and fat content, respectively. Four rats weighing about 50 g each were labeled with picric acid. One was used as a control group and three were injected with the miR-34c agomir. Surgery was performed under anesthesia minimizing animal pain, according to the criteria of the Committee of Ethics of our Institution. The research protocol was approved by the local Institutional Review Board to which the author belongs and was tested in accordance with the procedures and guidelines developed by the Animal Care and Use Committee of the Navy Medical University (Second Military Medical University), Shanghai, China.

### Masson's trichrome staining

Rat liver paraffin sections were dewaxed with tap and distilled water, and then stained with Regaud hematoxylin dye or Weigert hematoxylin for 5-10 min. The slides were stained with Masson Ponceau Acidic Red for 5-10 min. Then the slides were placed into 2% acetic acid dissolved in aqueous solution and later in 1% phosphomolybdic acid aqueous solution for 3-5 min. Afterwards, without washing, sections were counterstained with aniline blue or green dye for 5 min followed by 0.2% acetic acid aqueous. Finally, the dehydration step was performed with alcohol and xylene, and coverslips were sealed with gum.

### Sudan black staining

Fresh rat liver frozen 6-8 µm sections were cut and rinsed in 50%-70% alcohol for 20-30 s. Sections were then stained using Sudan Black B dye for 3-5 min, and again rinsed in 50%-70% alcohol for a few seconds and then washed with distilled water. Nuclei were counterstained with red dye solution for 5 min, washed for 1-2 min, and covered with glycerol gelatin.

### Statistical Analysis

SPSS v23.0 statistical software was used to analyze the results. Homogeneity of variance using one-way ANOVA (one-way ANOVA) was tested between groups (using LSD-T test). Kruskal-Wallis test was used when variance was not homogeneous; meanwhile values of each group were compared with each other by Nemenyi test. Statistical significance was considered when *p* < 0.05. Graphpad Prism v6.01 was used to draw charts.

## Results

### MiR-34c is significantly up-regulated but ACSL1 is down-regulated during natural activation of HSC

**Figure 1:**
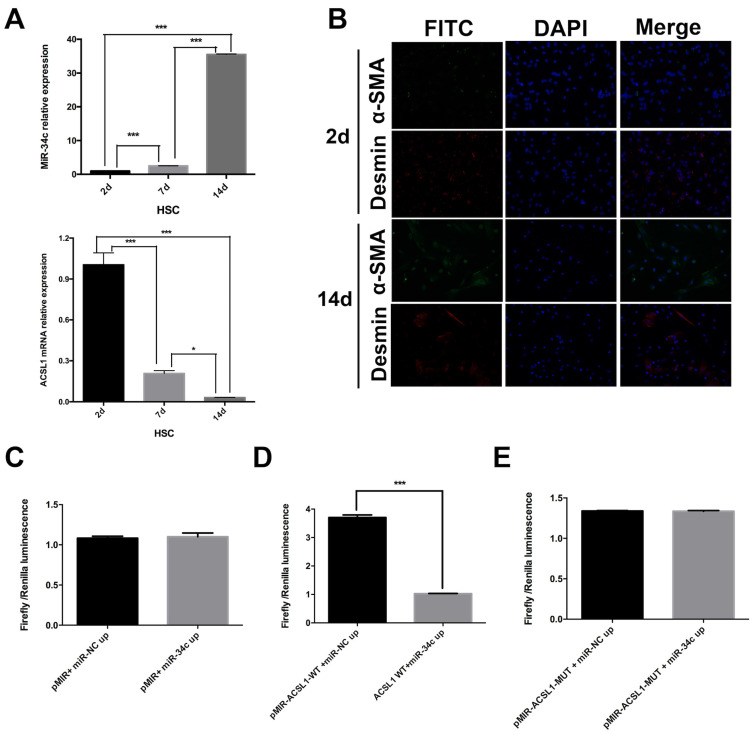
** miR-34c up-regulation and ACSL1 down-regulation are associated with HSC activation.** (**A**) The relative expression of miR-34c increased 47.6-fold in 14-day (activated) HSC compared to 2-day (stationary) HSC (*P* < 0.001). The relative expression of ACSL1 decreased 0.98-fold (*P* < 0.001) compared to the 2-day (stationary) HSC and 14-day (activated) HSC. (**B**) α-SMA and Desmin staining was primarily evident in HSC under different states of immunofluorescence. (**C**)There was no significant difference in the fluorescence intensity between the experimental group (pMIR+ miR-34c up) and the control group (pMIR+ miR-NC up) in cells overexpressing miR-34c (*P* = 0.781). (**D**) After modifying the binding site of miR-34c in the vector using the ACSL1 wild type plasmid in group pMIR+ACSL1-WT + miR-34c up, the fluorescence intensity in group pMIR+ACSL1-WT + miR-NC up was significantly different compared to the pMIR+ miR-NC up group (*P* < 0.001). (**E**) miR-34c could not bind to the mutant ACSL1 plasmid. There was no significant difference in fluorescence intensity between pMIR-ACSL1-MUT + miR-NC up and pMIR-ACSL1-MUT + miR-34c up (*P* = 0.952).

MiR-34c and ACSL1 expression were evaluated in primary HSC on days 2, 7 and 14. The experiment was repeated three times. Our results confirmed that the relative expression of miR-34c increased 47.6-fold in the 14-day treated HSC compared to quiescent HSC (2 days) (*P* < 0.001). Compared to the 7 day semi-activated HSC, the relative expression level of miR-34c in activated HSC was also dramatically different from that of quiescent and semi-activated HSC. Next, we measured the relative expression level of ACSL1 in semi-activated and activated HSC, which were significantly different from that of resting HSC (*P* < 0.001). The reduced ACSL1 expression was 98.1% in activated HSC relative to quiescent HSC (Fig 1A). Based on these results, these experiments show that ACSL1 expression was significantly reduced throughout HSC activation.

Additionally, we performed immunofluorescence to identify the status of HSC activation in culture using α-SMA and Desmin antibodies. The results showed that 2 day-cultured HSC (quiescent) are α-SMA negative in the cytoplasm, but Desmin positive in the cytoplasm. However, α-SMA and Desmin expression were expressed in the cytoplasm of cultured HSC for 14 days (Fig. 1B).

### A double fluorescence reporter assay: ACSL1 is a direct target gene of miR-34c

To determine whether ACSL1 is a target gene of miR-34c or not, we divided our cells into six groups. Each group was transfected with: pMIR+ miR-NC up, pMIR+ miR-34c up, pMIR-ACSL1-WT +miR-NC up; pMIR+ACSL1-WT + miR-34c up, pMIR-ACSL1-MUT + miR-NC up or pMIR-ACSL1-MUT + miR-34c up. There was no significant difference in the fluorescence intensity between group pMIR+ miR-NC up and pMIR+ miR-34c up (*P* = 0.781) (Fig. 1C). However, when the ACSL1 wild-type plasmid was used to combine with the binding site of miR-34c (pMIR+ACSL1-WT + miR-34c up) the fluorescence intensity was significantly different from that of experimental group pMIR-ACSL1-WT +miR-NC up (*P* <0.001) (Fig. 1D). No significant difference was found between pMIR-ACSL1-MUT + miR-NC up and pMIR-ACSL1-MUT + miR-34c up (*P* = 0.952) (Fig. 1E). Conclusively, these results suggest that ACSL1 is a direct target of miR-34c.

### Inhibition of miR-34C attenuates collagen synthesis and fibrogenesis in HSC-T6 cell line

**Figure 2:**
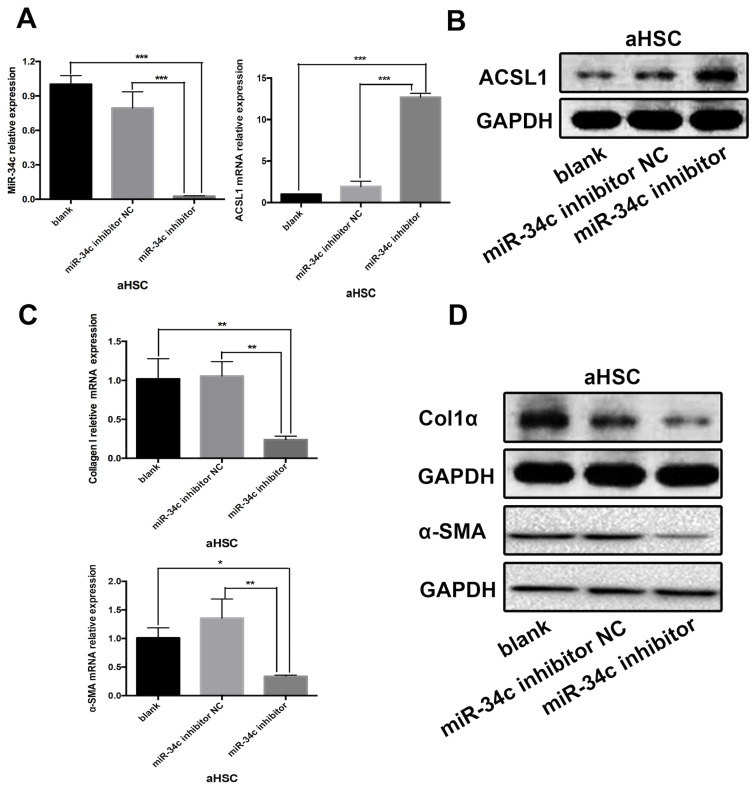
** Inhibition of miR-34C attenuates collagen synthesis in a HSC cell line.** (**A**) The relative expression levels of miR-34c in the blank and the control groups were significantly different compared to the experimental group (*P* < 0.001). Compared to the control group, the relative expression of ACSL1 in the experimental group increased 14.0-fold. The difference was statistically significant (*P* < 0.001). (**B**) ACSL1 protein expression in the experimental group was significantly higher compared to the blank and control groups. (**C**) Compared to the control group, the relative mRNA expression level of α-SMA in the experimental group decreased by 79.1%, and the difference was statistically significant (*P* < 0.01). Compared to the control group, the relative expression of Col1α in the experimental group was reduced by 81.8%, and the difference was statistically significant (*P* < 0.01). (**D**) Protein expression levels of α-SMA and Col1α in the experimental group were significantly lower compared to the blank and control groups.

Experiments were carried out in the HSC-T6 HSC cell line. The experimental design consisted of three groups: the blank group - untreated HSC-T6 cells; the control group - HSC-T6 cells transfected with miRNA inhibitor Negative Control; and the group of HSC-T6 cells transfected with the miR-34c inhibitor. This experiment was repeated three times. 48 h after transfection, the results of qRT-PCR showed that miR-34c inhibition significantly reduced miR-34c expression in the experimental group compared to the blank and control groups (Fig. 2A). Conversely, ACSL1 expression was higher compared to the blank and control groups (Fig. 2A, B). We measured protein expression of ACSL1-target genes, including α-SMA and Col1α using qRT-PCR and western blot analysis. In the miR-34c inhibition group, α-SMA and Col1α expression were significantly lower compared to the blank and control groups. (Fig. 2C, D). Our data indicate that inhibition of miR-34c attenuates the synthesis of collagen fibers in HSC cells.

### MiR-34c regulates ACSL1 expression and promotes initiation and development of liver fibrosis

**Figure 3:**
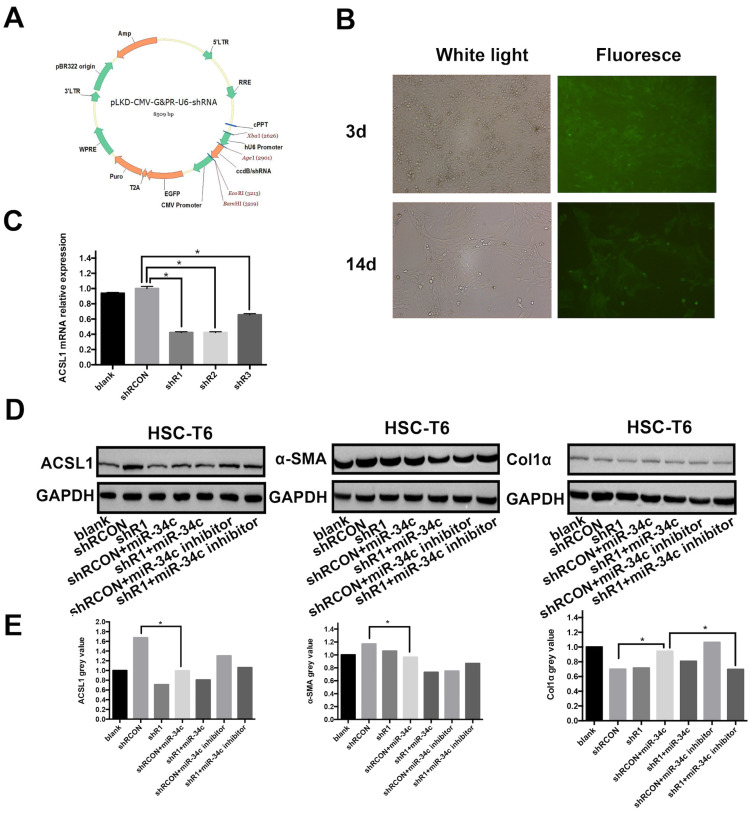
** MiR-34c activates and proliferates HSC by targeting ACSL1.** (**A**) Selected interference vectors pLKD-CMV-G&PR-U6-shRNA vector map. (**B**) Lentivirus infected HSC-T6 cells, screened for fluorescence of stable strains after 3 and 14 days. (**C**) ACSL1 mRNA expression in each group was reduced by: 57.76%, shR1 (*P* < 0.05); shR2 decreased by 57.67% (*P* < 0.05); and shR3 decreased by 34.49% (*P* < 0.05). (**D**) ACSL1, α-SMA, and Col1α protein expression in each group. The internal reference is GAPDH. (**E**) Gray scale analysis of the automated chemiluminescence imaging system indicated that the expression of ACSL1 in the shR1+miR-34c inhibitor group increased 1.49-fold, and the expression of α-SMA decreased by 18.22% and the expression of Col1α decreased by 2.58%.

The ccdB virulence genes downstream of the U6 promoter of vector pLKD-CMV-G & PR-U6-shRNA (Fig. 3A) were digested with Age I and EcoR I and inserted into the ACSL1 interference fragment to construct a plasmid that expresses green fluorescent protein EGFP and puromycin Rat ACSL1-resistant vector. qRT-PCR was used to detect mRNA expression of ACSL1 in three different interfering strains to understand the interference efficiency. The results showed that mRNA expression of ACSL1 in shR1, shR2 and shR3 decreased by 58%, 57% and 34%, respectively, compared to the control group. The *P* values were all < 0.05 and the differences were statistically significant (Fig. 3C). The results showed that shR1 interference stable cell line ACSL1 had the highest efficiency of interference. In the multiplicity of infection (MOI) of 200 conditions, HSC-T6 infection efficiency was approximately 80%. After 14 days of puromycin drug screening, the rate of HSC-T6 expressing GFP was beyond 80% and the fluorescence brightness was enhanced (Fig 3B). This indicates that the stable expression of HSC-T6 cell line pLKD-CMV-Puro-CMV-G&PR-ACSL1 (shR1) was successful. In this rescue experiment, ACSL1 protein expression was 1.49-fold higher compared to the shR1 group after transfection with the miR-34c inhibitor in the constructed low-expression ACSL1 stable cell line (Fig. 3C). Concomitantly, the expression of α-SMA and Col1α, markers of hepatic fibrosis, decreased by 18.22% and 2.58%, respectively (Fig. 3D). MiR-34c inhibition partly restored the function of ACSL1, further validating that miR-34c and promoting initiation and development of liver fibrosis by targeting ACSL1.

### MiR-34c promotes hepatic fibrosis and decrease liver lipid accumulation in rats and HSC-T6 cell line

**Figure 4:**
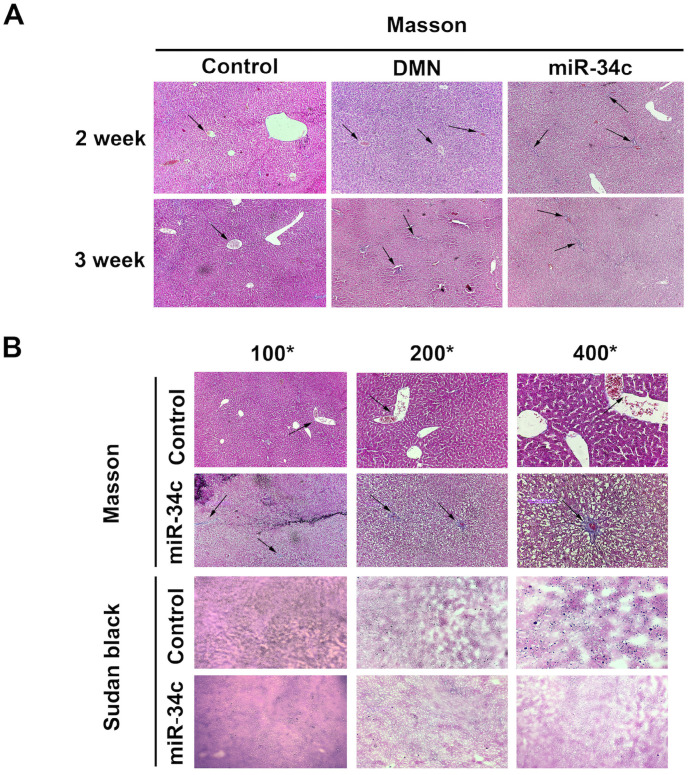
** MiR-34c agomir promotes hepatic fibrosis and inhibits liver lipogenesis in rats.** (**A**) Liver Masson trichrome staining in the normal control group, DMN model group, DMN model group, and the administration group was 100× for 2 weeks and 3 weeks; arrows point to the liver fibrosis areas. (**B**) Hepatic Masson trichrome stain and Sudan black stain in the DMN model group + administration group and normal control group at 4 weeks after administration. 100×, 200×, 400×, arrows indicate liver fibrosis areas.

**Figure 5:**
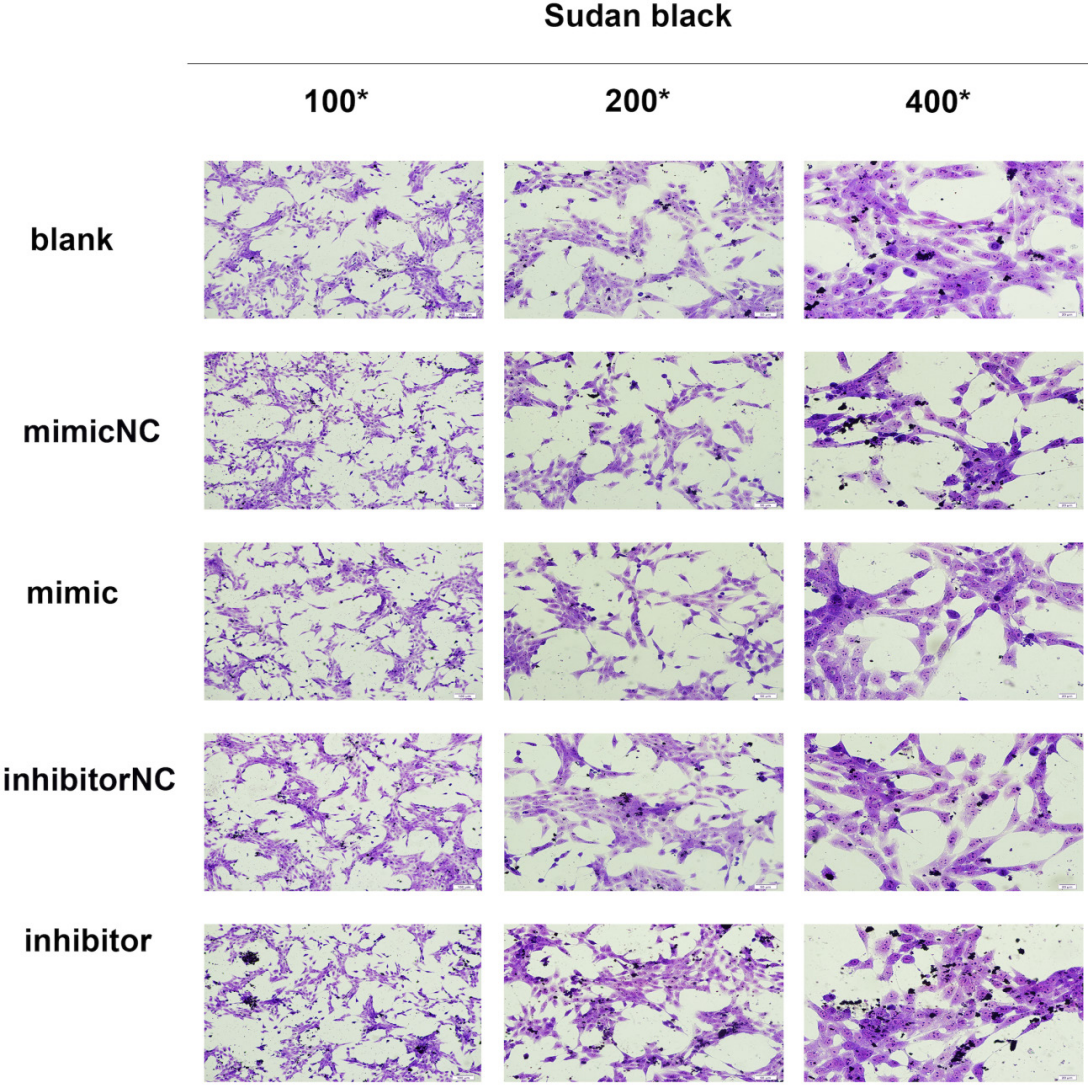
The content of lipid changes after transfected with MiR-34c mimic and inhibitor. HSC-T6 Sudan black staining in the blank group, normal control group, miR-34c mimic and inhibitor group, brownish black or dark black deposits are lipids, 100×, 200×, 400×.

In the current study, we established a model of early liver fibrosis using DMN in rats. Compared with the DMN model group, the degree of hepatic fibrosis and collagen fiber accumulation and deposition increased in rats treated with DMN and miR-34c agomir (Fig. 4A). In addition, Sudan black staining shows that the amount of lipid droplets in the miR-34c agomir group decreased compared to the blank control group (Fig. 4B). Moreover, we transfected miR-34c mimic and inhibitor in HSC-T6 cells. By Sudan black staining method, we found that lipid droplets were decreased in miR-34c mimic group. Conversely, lipid droplets were increased in miR-34c inhibitor group (Fig. 5A).

## Discussion

Recently, there are some studies have demonstrated that abnormal expression of miRNAs in liver tissue are related to the pathogenesis of liver disease, including viral hepatitis, liver fibrosis, and hepatocellular carcinoma (HCC) 23-26. Moreover, alteration of miRNAs target genes involved in hepatic energy metabolism, inflammatory, cell regeneration and fibrogenic signaling that drive the progression of nonalcoholic fatty liver disease (NAFLD) to liver fibrosis 27. However, the mechanisms coordinating the interaction between the different signaling pathways of miRNAs regulating HSC activation and hepatic fibrosis have not been investigated in great detail.

In the current study, we explored whether miR-34c can directly targets ACSL1 or not and further investigated its role in HSC activation and hepatic fibrogenesis. First, we successfully extracted and cultured HSC. The expression of miR-34c was significantly increased during the natural activation of HSC, while ACSL1 was significantly decreased. This is consistent with our previous study in an animal model of liver fibrosis which showed that miR-34c expression increased along with liver fibrosis. Next, we confirmed that ACSL1 is indeed a direct target of miR-34c using a double fluorescent reporter system. We also found inhibition of miR-34c attenuates collagen synthesis in HSC_T6 cell line. By inhibiting miR-34c function in HSC, ACSL1 expression was significantly increased. This mechanism was associated with attenuated HSC activation and decreased hepatic fibrosis. Next, we used an inhibitor of miR-34c in a stable low expression ACSL1 cell line. The expression of ACSL1 at the protein level increased 1.49-fold, whereas the expression of α-SMA and Col1α in liver fibrosis decreased by 18.22% and 2.58%, respectively. Our results showed that miR-34c inhibition partly restored the function of ACSL1, further validating that miR-34c targets ACSL1, thus regulating HSC activation and hepatic fibrogenesis. ACSL on the endoplasmic reticulum and mitochondrial outer membrane catalyzes the formation of acyl-CoA with FA of 12-20 carbon atoms, which are lipid metabolism intermediates and are involved in FA metabolism, membrane, modification, and several physiological processes 28, 29. Besides, ACSL1 is critical in hepatic lipid homeostasis and is considered to play an essential role in activating FA synthesis of triglyceride (TG) 30, 31. These indicate that ACSL1 is closely related to FA and lipids. It has been reported that saturated FA, such as oleic acid (OA) and palmitic acid (PA), also participate in myofibroblast and HSC activation 21, 32.

To further investigate the regulation of miR-34c to hepatic fibrosis and the variety of lipid *in vivo*, we developed a rat model of early liver fibrosis using DMN models. Compared to the DMN model group, the degree of hepatic fibrosis increased in animals treated with a combination of DMN and miR-34c agomir, indicating that miR-34c promotes hepatic fibrogenesis which was in accordance with the results in activated HSC. Our findings suggested that miR-34c might play a promoting role in liver fibrogenesis, and its overexpression might be associated with the HSC activation. Moreover, we found a phenomenon that Sudan black staining revealed that the number of lipid droplets in the liver decreased in the miR-34c agomir group. However, so far, how miR-34c affects intrahepatic lipid content and thus affects liver fibrosis remains elusive. The liver is made up of many cell types 33. But we don't know that lipid reduction in the liver occurs in which kind of cell. To further verify whether lipid droplets were associated with hepatic stellate cells or not which are closely related to liver fibrosis, we validated in a rat hepatic stellate cell line (HSC-T6). After transfected with miR-34c mimic and inhibitor, the result was consistent with *vivo* data by Sudan black staining. In all, we found out that miR-34c agomir can promote liver fibrosis and reduce lipid depositon in the liver. Both animal experiments and cell experiments verified miR-34c could affect liver fibrosis and lipid of hepatocytes and HSC.

In the liver, sustained TG and its hydrolysate FA, accretion leads to NAFLD, eventually progressing to NASH and cirrhosis 34. More and more reports that a minority of NAFLD patients can lead to progressive nonalcoholic steatohepatitis (NASH), fibrosis, and ultimately HCC 35-37. Our research has shown that miR-34c activates and proliferates HSC to further promote liver fibrosis by targeting ACSL1. Currently, Li and colleagues found that the miR-34 family participates in the process of hepatic fibrosis by regulating the Peroxisome proliferator-activated receptor γ (PPARγ) pathway 38. PPAR-γ and PPAR-α can regulate ACSL1 and increase the expression of genes involved in free FA formation and TG levels 31, 39, 40. Another study shows that miR-34a can further regulate hepatic lipid metabolism by directly targeting PPARα signaling 41. When we injected the 34c agomir, ACSL1 was significantly inhibited *in vivo* resulting in lipid content in the liver is affected and the FA synthesis of hepatocytes in the liver was changed, which significantly reduced the lipid droplets in whole liver by Sudan black staining. The cellular lipidtartalom was reduced of miR-34c mimic group and was accumulated of miR-34c inhibitor group, which may be due to the fact that the miR-34c agomir suppressed the expression of ACSL1 and thus affected lipid synthesis in the liver. The process of our findings may be achieved by miR-34c targeting ACSL1 and altered regulation of the PPARγ pathway. Given the accumulation of lipid in the liver, it was expected to play an important role in HSC activation and hepatic fibrosis. Therefore, it is necessary to further study the regulation of ACSL1 to lipid and the role of lipid in the development of hepatic fibrosis, it is of utmost interest to find new breakthroughs in the mechanisms of liver fibrogenesis and its reversal.

In summary, using functional experiments with HSC and *in vivo* experiments in rats, we further confirmed that miR-34c promotes development of hepatic fibrosis by targeting ACSL1. When miR-34c function was inhibited, HSC activation was inhibited, ACSL1 expression increased, and α-SMA and Col1α expression decreased. Our data confirmed that injection of the miR-34c agomir increased the degree of fibrosis but strongly attenuated lipidtartalom. Therefore, we hypothesize that the lipid metabolism pathway may be regulated by miR-34c inhibition, thus improving the course of liver fibrosis. Our results open a new therapeutic avenue/offer a promising way for the prevention and efficacious treatment strategies for hepatic fibrosis by investigating how miRNAs are involved in HSC activation and reversing hepatic fibrosis.

## Figures and Tables

**Table 1:** Primer sequences

Gene (mouse)	Primer sequences (5'- 3')
ACSL1	Forward: TGTCCATGGAGAAAGCTTG
	Reverse: GATATCCTTGTTCCTGCACAG
GAPDH	Forward: ACAGCAACAGGGTGGTGGAC
	Reverse: TTTGAGGGTGCAGCGAACTT
α-SMA	Forward: TTCCTTCGTGACTACTGCTGAG
	Reverse: CAATGAAAGATGGCTGGAAGAG
Col1α	Forward: ATGTCTGGTTTGGAGAGAGCA
	Reverse: GAGGAGCAGGGACTTCTTGAG
U6	Forward: GCAGTGCTTAGCTGGTTGT
	Reverse: GCGAGCACAGAATTAATACGAC
miR-34c	Forward: CGCGGATCCTGCTGCGTGCTGTGATTC
	Reverse: GTGGAATTCTTTCCCTGTGGCTGTCCTC

## Abbreviations

HSC: hepatic stellate cells****
ACSL1: acyl-CoA synthetase long-chain family member-1
FA: fatty acid
DMN: dimethylnitrosamine
miRNAs: microRNAs
α-SMA: α-smooth muscle actin
Col1α: type I collagen
DMEM: dulbecco modified eagle medium
FBS: fetal bovine serum
HCC: hepatocellular carcinoma
NAFLD: nonalcoholic fatty liver disease
TG: triglyceride
OA: oleic acid
PA: palmitic acid
NASH: nonalcoholic steatohepatitis
PPARγ: peroxisome proliferator-activated receptor γ

## Innovation & Noteworthy

This is the first research showing miR-34c to have a regulatory effect on HSC activation and promotes hepatic fibrosis by targeting ACSL1, a key enzyme related to fatty acid and lipid synthesis closely. MiR-34c increased the degree of fibrosis but attenuated lipid accumulation. As a new target of gene therapy, miRNA is gradually showing its potential clinical application, our results open a new therapeutic avenue for the prevention and treatment of hepatic and a method of reversing hepatic fibrosis.
